# Association between Insulin-Like Growth Factor 1 Gene rs12423791 or rs6214 Polymorphisms and High Myopia: A Meta-Analysis

**DOI:** 10.1371/journal.pone.0129707

**Published:** 2015-06-15

**Authors:** Lan Guo, Xueying Du, Ciyong Lu, Wei-Hong Zhang

**Affiliations:** 1 Department of Medical statistics and Epidemiology, School of Public Health, Sun Yat-sen University, Guangzhou, 510080, People’s Republic of China; 2 Department of laboratory, Center for the primary and secondary school health promotion, Guangzhou, 510080, People’s Republic of China; 3 Epidemiology, Biostatistics and Clinical Research Centre, School of Public Health, Université Libre de Bruxelles (ULB), Belgium; University of Utah, UNITED STATES

## Abstract

**Objective:**

To evaluate the association of insulin-like growth factor 1 gene rs12423791 and rs6214 polymorphisms with high myopia.

**Methods:**

An electronic search was conducted on PubMed, Embase, the Cochrane Library and the Chinese Biological Abstract Database for articles published prior to May 6, 2014. A meta-analysis was performed using Revman 5.1 and Stata 12.0, and the odds ratios with 95% confidence intervals were calculated in fixed or random effects models based on the results of the Q test. The subgroup analysis was conducted on the basis of the various regions, the sensitivity analysis was also performed to evaluate the stability of the results, and the publication bias was evaluated by a funnel plot and Egger’s linear regression analysis.

**Results:**

This comprehensive meta-analysis included 2808 high myopia patients and 2778 controls from five unrelated studies. The results demonstrated that the significant association was not present in any genetic models between IGF-1 rs12423791 or rs6214 and high myopia. However, subgroup analysis indicated that rs12423791 polymorphism was associated with high myopia in the Chinese populations in the allelic contrast model (C vs. G: OR=1.24, 95% CI=1.04-1.48 in the fixed-effects model), the dominant model (CC+CG vs. GG: OR=1.40, 95% CI=1.16-1.69 in the fixed-effects model), and the codominant model (CG vs. GG: OR=1.37, 95% CI= 1.12-1.68 in the fixed-effects model). Additionally, none of the individual studies significantly affected the association between IGF-1 rs12423791 and high myopia, according to sensitivity analysis.

**Conclusion:**

This meta-analysis shows that IGF-1 rs12423791 or rs6214 gene polymorphism is not associated with high myopia.

## Introduction

Myopia is a complex disease caused by multiple genetic and environmental factors and potentially the interaction of those two factors. [1] It affects both children and adults and has an average prevalence of 30% worldwide. [2,3] Myopia is a refractive error of the eye and is associated with vision impairments including glaucoma and retinal detachment. [4,5] High myopia is usually defined as a refractive error of -6.00 diopters (D) or less and without other known ocular or systemic diseases. [6] It has been reported that high myopia is a public health problem internationally, imposing an enormous economic and social burden on many countries, and China is no exception. [7,8,9] High myopia is now considered to be the fourth most common cause of irreversible blindness. [10] Thus, it is very important to identify the risk factors for high myopia and to establish preventive strategies for high myopia.

Previous studies have demonstrated that environmental exposure is a risk factor for myopia; for example, outdoor activities have been increasingly recognized as protective factors for myopia. [11,12] While the exact mechanism of myopia formation is still unclear, there is also genomic evidence from different ethnic populations, including twin studies, that demonstrate that genetics play an important role in the development of myopia, [13,14] especially high myopia. [15] Additionally, many genome-wide association studies for high myopia have also found or identified many candidate genes and loci for high myopia, [16,17,18] and a genome-wide meta-analysis of myopia also provides evidence in favor of replication of 11 loci involved in causation of myopia. [19]

Insulin-like growth factor-1 (IGF-1) is a member of the human growth hormone-insulin-like growth factor pathway that plays a key role in growth and metabolism. It can also regulate scleral proteoglycan production, [20] potentially by upregulating gene transcription, translation, or the activation of sulfotransferases, which in turn increases the synthesis of sulfated proteoglycans. [21] Previous animal studies demonstrated that IGF-1 contributes to eye growth and myopia development. [22,23] In addition, the IGF-1 gene is located on chromosome 12q23.2 and is within the MYP3 interval that has been mapped for autosomal dominant high myopia. [24] Recently, many genetic studies showed that rs12423791 or rs6214 polymorphisms in IGF-1 were significantly associated with high or extreme myopia in Caucasian and Chinese populations; [25,26] however, another study demonstrated that IGF-1 gene rs12423791 and rs6214 polymorphisms were not associated with high myopia, [27,28] even though a number of single nucleotide polymorphisms (SNPs) in IGF-1 have been reported. Therefore, we performed a meta-analysis to examine whether there is such an association and to produce a reliable estimate of the association between IGF-1 gene rs12423791 and rs6214 polymorphisms and high myopia.

## Methods

### Search strategy

To assess the complete evidence of an association between the IGF-1 gene and high myopia, we performed the present comprehensive meta-analysis of published studies. An electronic search was conducted on PubMed, Embase, the Cochrane library and the Chinese Biological Abstract Database for articles published prior to May 6, 2014. The search strategy was based on a combination of ‘(IGF-1, insulin-like growth factor 1) and (gene or variants or polymorphism or alleles or mutation) and (myopia, high myopia)’ without language restrictions. The references of the retrieved articles were also screened.

### Selection criteria

Inclusion and exclusion criteria: all the studies that were included satisfied all of the following criteria: 1. the studies evaluated the rs12423791 and rs6214 polymorphisms in the insulin-like growth factor 1 gene and high myopia; 2. the studies including normal individuals with spherical refraction ranged from -1.5 to 1.5 D and free from any complications as control subjects, and high myopia was defined as the axial length of 26 mm or higher and/or a refractive error of -8.0 D or less; 3. the studies contained sufficient published data to estimate an odds ratio (OR) and a 95% confidence interval (CI); 4. the studies provided genotype or allele distributions in both the case and control groups; 5. the studies were independent studies; 6. the studies presenting non-original data, such as reviews, editorials, opinion papers, or letters to the editor, were excluded; 7. the studies using nonhuman subjects or specimens were excluded; and 8. the studies with no extractable numerical data were excluded.

### Data extraction

Two researchers independently applied the inclusion criteria to all identified studies and made decisions on which studies to include. Differences were resolved by discussion and consultation with a third author. The following data were extracted from each study: the first author, year of publication, study design, study population, definition of high myopia, number or frequency by different genotypes, number of cases and controls, minor allele frequency (MAF) of cases and controls, mean age, mean spherical equivalent and mean axial length of high myopia participants.

### Ethical Statement

This study was approved by the ethics committee of the School of Public Health of Sun Yat-sen University. Then the researches were allowed to read and analyze the data.

### Statistical analysis

The odds ratios (ORs) with 95% confidence intervals (CIs) were computed to assess the strength of the association between IGF-1 gene polymorphisms and high myopia risk. The significance of the pooled ORs was determined by the Z-test, with a *P*<0.05 considered statistically significant. We calculated the Q statistic to estimate the heterogeneity, and a *P*≤0.10 was considered statistically significant for the Q-statistic test. The I^2^ statistic was used to quantify heterogeneity, and an I^2^-value of 0% indicated no observed heterogeneity with larger values showing increased heterogeneity. [29] If heterogeneity existed among the studies, a random-effects model was used to compute the summary risk estimate; if there was no heterogeneity, a fixed-effects model was used. [30] In addition, we assessed whether the genotype frequencies of the controls in the individual studies were consistent with the expected distribution, that is, in Hardy-Weinberg equilibrium (HWE); a *P*<0.05 was considered statistically significant in this test. Because the potential cause of heterogeneity among studies was geographic region, the subgroup analysis was conducted on the basis of the various regions. To test the stability of the association, we performed a one-way sensitivity analysis by excluding each study in the meta-analysis in turn. The possibility of publication bias was assessed by visual inspection of a funnel plot in which the standard error of the log (OR) of each study was plotted against its corresponding log (OR); an asymmetric plot indicates possible publication bias. Additionally, Egger’s linear regression test was used to evaluate asymmetry, and a *P*<0.05 was set as significant. All statistical analyses were performed using RevMan 5.1 (Revman; The Cochrane Collaboration, Oxford, UK) and Stata 12.0 (StataCorp, The College Station, Texas, USA).

## Results

### Eligible studies and study characteristics

**Fig 1:**
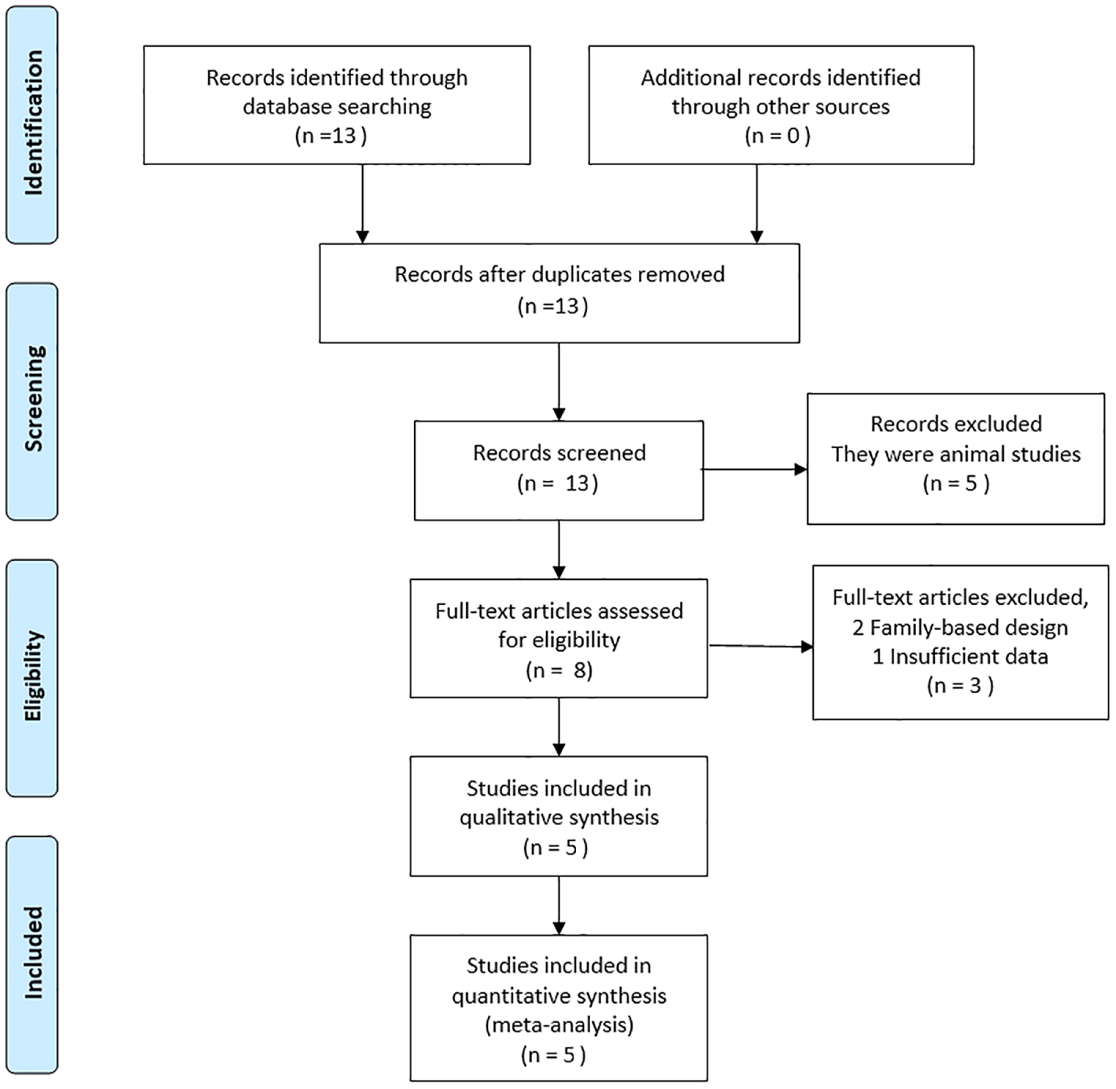
Flow chart of the study selection process. Literature search flow.

A total of thirteen potentially relevant studies were retrieved. Of these, five studies were excluded because they were animal studies, which were determined by reading the title and abstract. After a more detailed full-text review, three studies were excluded because two used a family-based design and the other failed to extract sufficient data. Some IGF-1 gene SNPs, such as rs10860861 and rs5742632, were described in only one or two studies. To ensure the validity of the results, any IGF-1 gene polymorphisms reported in fewer than four studies were not included. Thus, only rs12423791 and rs6214 were analyzed in our meta-analysis. Finally, five studies including rs12423791 and rs6214 were included in this meta-analysis. A detailed flow chart of the study selection is shown in Fig 1. In total, there were 2808 participants with high myopia and 2778 controls without high myopia or other ocular diseases. The years of publication ranged from 2011 to 2013, and the populations involved were Chinese and Japanese. The Hardy-Weinberg test (HWE) was performed on all of the included studies, and the results showed that the IGF-1 gene genotype frequencies of all five studies were in HWE in the controls. (Table 1)

**Table 1:** Basic characteristics of the included studies.

Authors	Ref.No.	Populations	Participants		Mean age (y)		Mean SRE (D)	Mean Axial length (mm)	rs12423791 MAF		rs6214 MAF		Definiton of high myopia	HWE
			High myopia	Controls	High myopia	Controls	High myopia*	High myopia*	Cases	Controls	Cases	Controls		
Mak, 2012	33	Chinese	300	300	27.6	24.6	-10.53±2.48	27.76±1.13	0.317	0.325	0.487	0.512	SRE≤-8.00 D	yes
Zhuang, 2012	23	Chinese	421	401	38.29±16.57	68.77±10.65	-14.57±5.60	28.33±2.26	0.218	0.229	0.479	0.499	SRE≤-8.00 D	yes
Miyake, 2013	24	Japanese	1339	1194	57.20±14.90	50.30±15.90	-12.69±4.54	29.18±1.85	0.265	0.267	0.463	0.469	AL≥26 mm	yes
Yoshida, 2013	25	Japanese	446	481	37.9±11.90	39.3±11.0	-11.7±2.24	28.0±1.16	0.266	0.305	0.359	0.338	SRE≤-9.00 D	yes
Zhao, 2013	34	Chinese	302	402	41.24±16.34	43.32±22.15	-16.54±5.26	28.98±2.27	0.296	0.229	0.465	0.499	SRE≤-8.00 D	yes

Abbreviations: Ref: reference; SRE: spherical refractive errors; y: year; D: diopter; MAF: minor allele frequency; AL: axial length; HWE: Hardy-Weinberg equilibrium.

*The measurement data was of the right eye.

### Association between IGF-1 gene rs12423791 and high myopia

**Fig 2:**
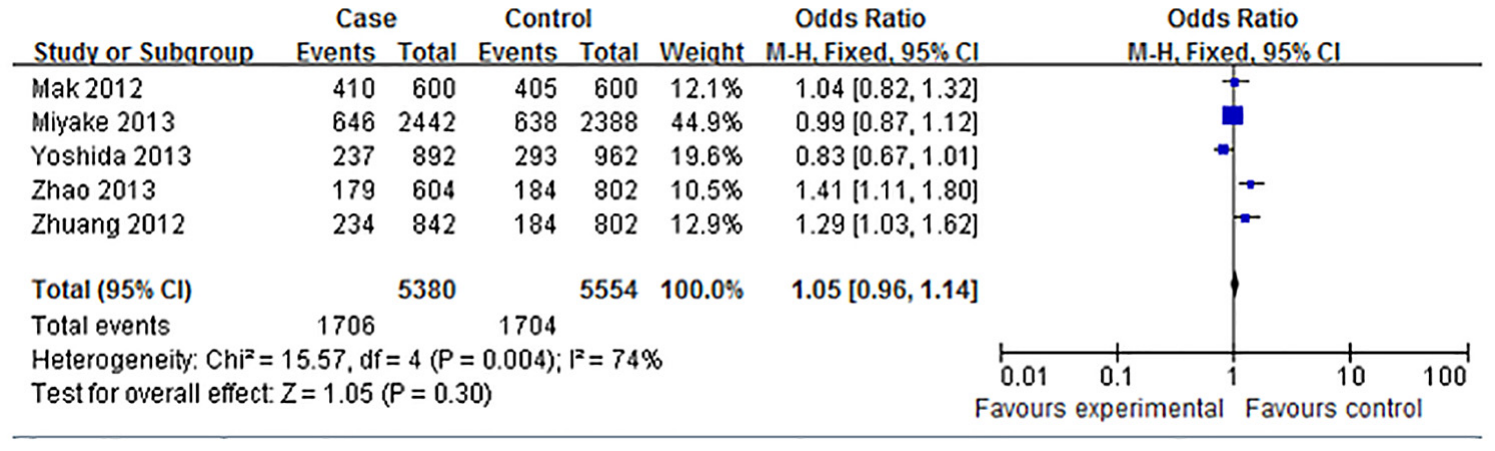
Forest plots of the pooled ORs with 95% CIs for associations between IGF-1 rs12423791 and high myopia in allelic contrast model (C vs. G). Events: the number of individuals carrying the C allele.

**Fig 3:**
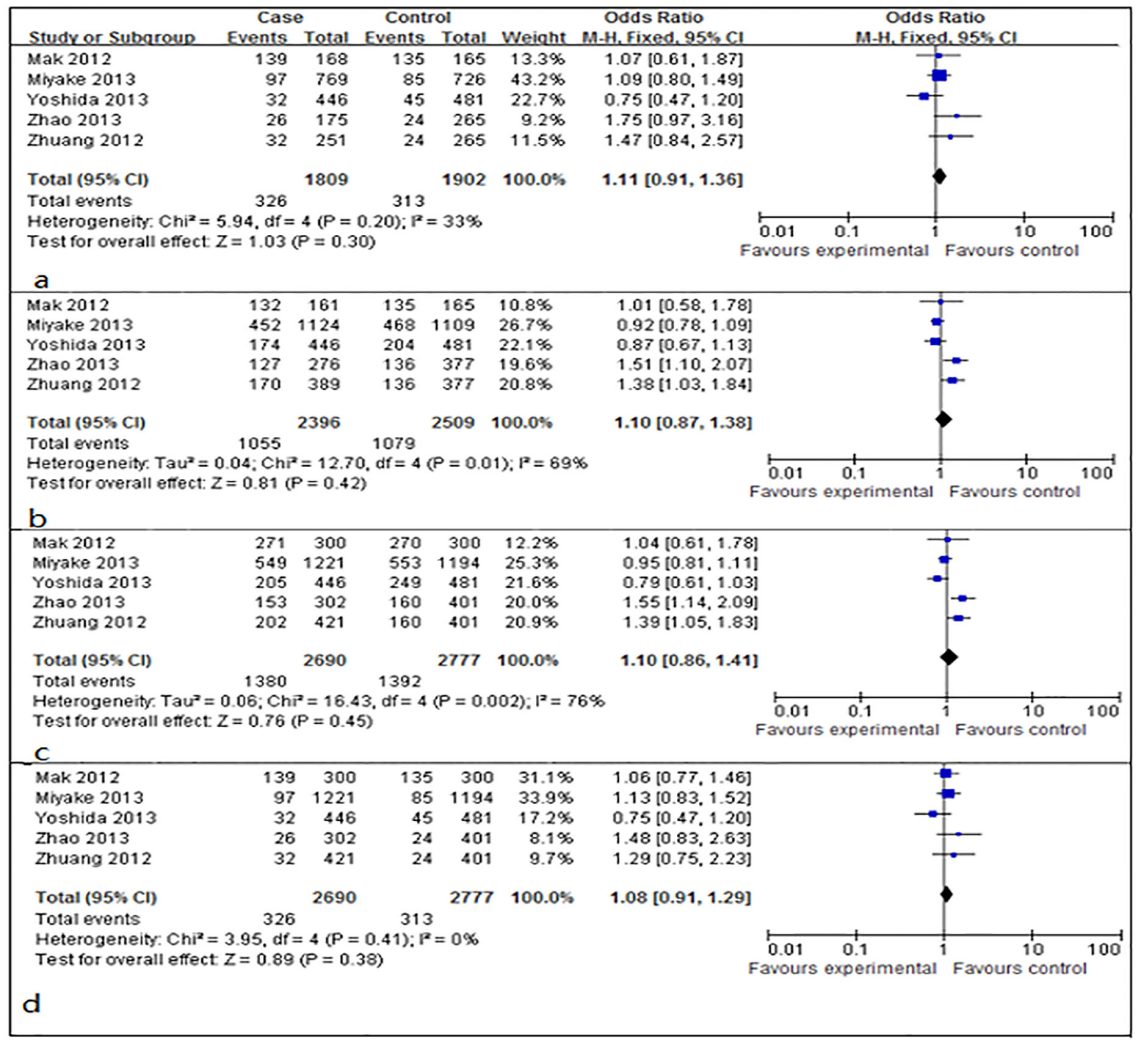
Forest plots of the pooled ORs with 95% CIs for associations between IGF-1 rs12423791 and high myopia in codominant model, dominant model, and recessive model. (a) Codominant model (CC vs. GG); events: the number of CC genotype in a. (b) Codominant model (CG vs. GG); events: the number of CG genotype in b. (c) Dominant model (CC+CG vs. GG); events: the number of CC+CG genotype in c. (d) Recessive model (CC Vs. CG+GG); events: the number of CC genotype in d.

Allele frequencies of rs12423791 were determined in the cases and controls. As shown in Figs 2 and 3, the Q test suggested that there was significant between-study heterogeneity in the allelic contrast model (C vs. G, I^2^ = 74%, *P* = 0.004), the dominant model (CC+CG vs. GG, I^2^ = 76%, *P* = 0.002), and the codominant model (CG vs. GG, I^2^ = 69%, *P* = 0.01), then the random-effect models were used to calculate the pooled ORs in these models. The fixed-effect models were used in the codominant model (CC vs. GG, I^2^ = 33%, *P* = 0.20) and recessive model (CC vs. CG+GG, I^2^ = 0%, *P* = 0.41). The results demonstrated that no significant associations between IGF-1 rs12423791 and high myopia were present in the allelic model (OR: 1.05, 95% CI: 0.96–1.14, *P*>0.05 in the random-effects model), the codominant model (CC vs. GG: OR = 1.11, 95% CI = 0.91–1.36, *P*>0.05 in the fixed-effects model; CG vs. GG: OR = 1.10, 95% CI = 0.87–1.38, *P*>0.05, in the random-effects model), the dominant model (CC+CG vs. GG: OR = 1.10, 95% CI = 0.86–1.41, *P*>0.05 in the random-effects model), and the recessive model (CC vs. CG+GG: OR = 1.08, 95% CI = 0.91–1.29, *P*>0.05 in the fixed-effects model). (Table 2)

**Table 2:** Main results of the pooled ORs in the meta-analysis.

SNPs	Inheritance Model		OR	95% CI	*P*	*P* (Q test)*
**rs12423791**						
	Allelic contrast model	C vs. G	1.05	0.96–1.14	0.30	0.004
	Codominant model	CC vs. GG	1.11	0.91–1.36	0.30	0.20
		CG vs. GG	1.10	0.87–1.38	0.42	0.01
	Dominant model	CC+CG vs. GG	1.10	0.86–1.41	0.45	0.002
	Recessive model	CC vs. CG+GG	1.08	0.91–1.29	0.38	0.41
**rs6241**						
	Allelic contrast model	A vs. G	1.02	0.94–1.10	0.68	0.42
	Codominant model	AA vs. GG	1.04	0.90–1.21	0.59	0.51
		AG vs. GG	1.04	0.90–1.19	0.59	0.99
	Dominant model	AA+AG vs. GG	1.04	0.91–1.18	0.55	0.88
	Recessive model	AA vs. AG+GG	1.01	0.90–1.13	0.87	0.39

*The Q statistic was used to estimate heterogeneity, and a *P*≤0.10 was considered statistically significant for the Q-statistic test.

Further subgroup analysis by study population indicated that rs12423791 polymorphism was associated with high myopia in the Chinese population in the allelic contrast model (C vs. G: OR = 1.24, 95% CI = 1.04–1.48 in the fixed-effects model), the dominant model (CC+CG vs. GG: OR = 1.40, 95% CI = 1.16–1.69 in the fixed-effects model), the codominantmodel (CG vs. GG: OR = 1.37, 95% CI = 1.12–1.68 in the fixed-effects model). (Table 3)

**Table 3:** Results of the subgroup analysis by study population.

Study population	SNPs	Inheritance Model	OR	95% CI	P	*P*(Q test)*
**Chinese**						
	**rs12423791**					
		C vs. G	1.24	1.04–1.48	0.02	0.19
		CC vs. GG	1.39	1.00–1.93	0.05	0.48
		CG vs. GG	1.37	1.12–1.68	0.002	0.48
		CC+CG vs. GG	1.40	1.16–1.69	<.001	0.45
		CC vs. CG+GG	1.17	0.91–1.50	0.21	0.56
	**rs6241**					
		A vs. G	1.05	0.92–1.19	0.50	0.30
		AA vs. GG	1.10	0.87–1.40	0.43	0.37
		AG vs. GG	1.05	0.85–1.29	0.68	0.99
		AA+AG vs. GG	1.06	0.87–1.30	0.54	0.80
		AA vs. AG+GG	1.07	0.88–1.30	0.48	0.24
**Japanese**						
	**rs12423791**					
		C vs. G	0.92	0.78–1.09	0.33	0.15
		CC vs. GG	0.97	0.75–1.26	0.83	0.20
		CG vs. GG	0.91	0.79–1.04	0.17	0.71
		CC+CG vs. GG	0.89	0.76–1.05	0.18	0.25
		CC vs. CG+GG	1.00	0.78–1.29	0.99	0.15
	**rs6241**					
		A vs. G	0.99	0.90–1.10	0.91	0.29
		AA vs. GG	1.01	0.83–1.22	0.96	0.33
		AG vs. GG	1.03	0.86–1.24	0.73	0.56
		AA+AG vs. GG	1.02	0.86–1.21	0.79	0.41
		AA vs. AG+GG	0.98	0.84–1.13	0.74	0.41

*The Q statistic was used to estimate heterogeneity, and a *P*≤0.10 was considered statistically significant for the Q-statistic test.

### Association between IGF-1 gene rs6214 and high myopia

**Fig 4:**
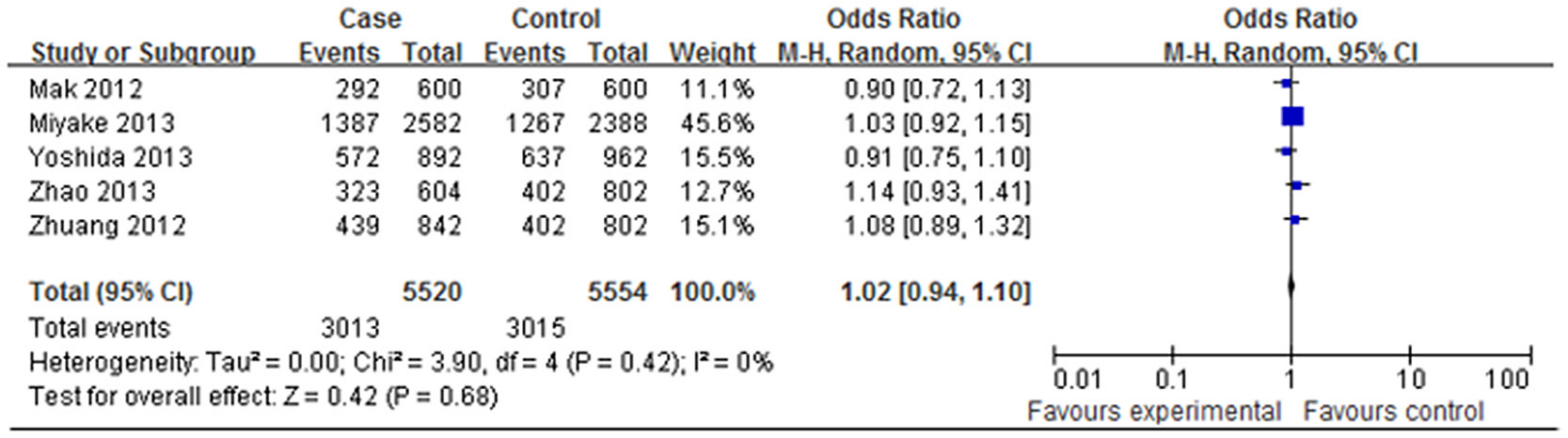
Forest plots of the pooled ORs with 95% CIs for associations between IGF-1 rs6214 and high myopia in allelic contrast model (A vs. G). Events: the number of individuals carrying the A allele.

**Fig 5:**
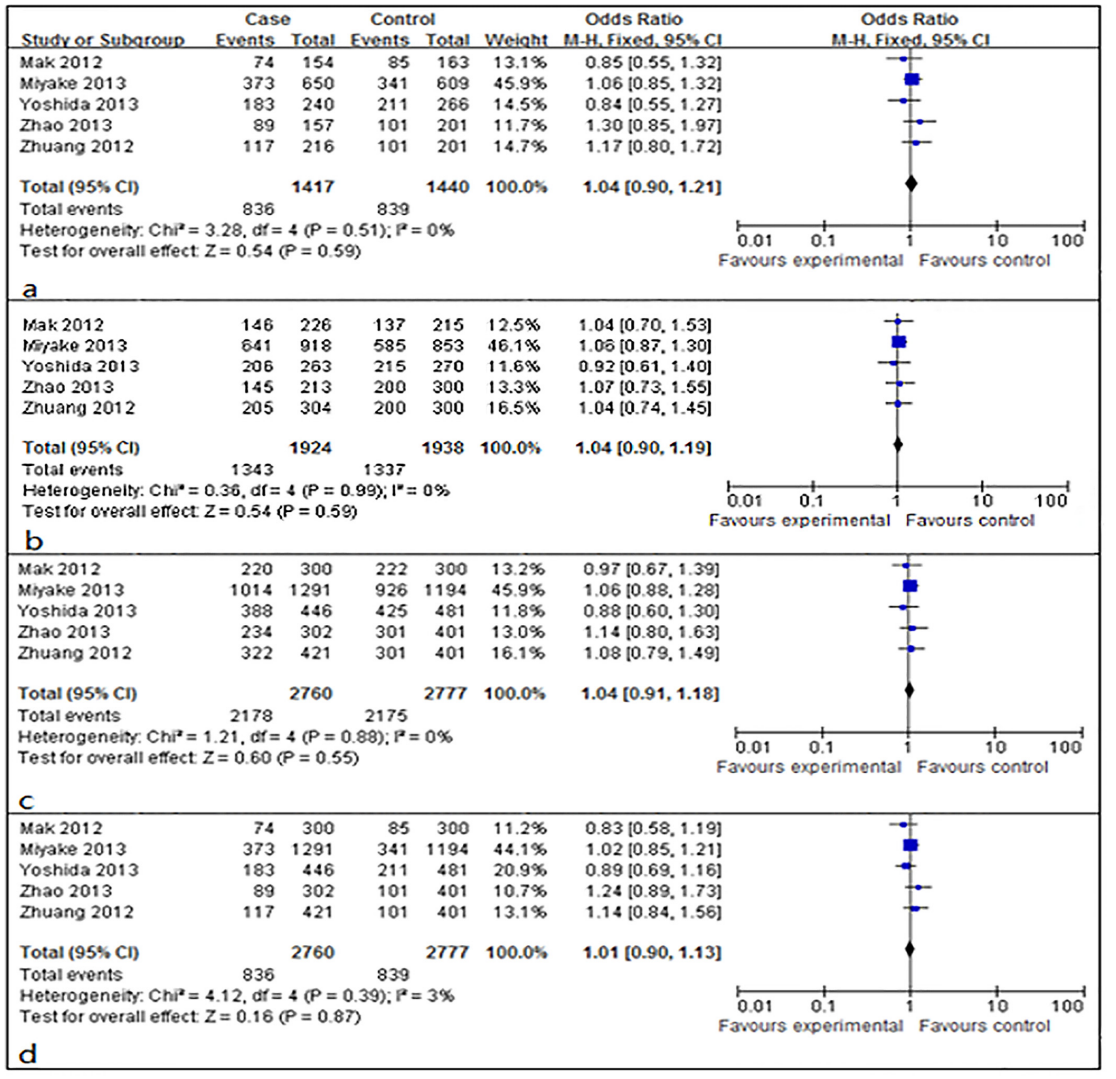
Forest plots of the pooled ORs with 95% CIs for associations between IGF-1 rs6241 and high myopia in codominant model, dominant model, and recessive model. (a) Codominant model (AA vs. GG); events: the number of AA genotype in a. (b) Codominant model (AG vs. GG); events: the number of AG genotype in b. (c) Dominant model (AA+AG vs. GG); events: the number of AA+AG genotype in c. (d) Recessive model (AA vs. AG+GG); events: the number of AA genotype in d.

Allele frequencies of rs6214 were determined in the cases and controls. As shown in Figs 4 and 5, the Q test did not show significant between-study heterogeneity in all of the comparison models (*P*>0.10). The fixed-effects models were used to calculate the pooled ORs, and the results suggested that there were not significant associations in the allelic contrast model (A vs. G: OR = 1.02, 95% CI: 0.94–1.10, *P*>0.05 in the fixed-effects model), the codominant model (AA vs. GG: OR = 1.04, 95% CI = 0.90–1.21, *P*>0.05 and AG vs. GG: OR = 1.04, 95% CI = 0.90–1.19, *P*>0.05 in the fixed-effects model), the dominant model (AA+AG vs. GG: OR = 1.04, 95% CI = 0.91–1.18, *P*>0.05 in the fixed-effects model), and the recessive model (AA vs. AG+GG: OR = 1.01, 95% CI = 0.90–1.13, *P*>0.05 in the fixed-effects model). (Table 2) Further subgroup analysis by study population also could not find any associations between rs6214 and high myopia. (Table 3)

### Sensitivity analysis

A sensitivity analysis was conducted to explore the source of this heterogeneity. In our study, we only performed sensitivity analyses to assess the influence of each study on the IGF-1 gene allelic contrast model (C vs. G), dominant model (CC+CG vs. GG), and codominant model (CG vs. GG), because there was not significant heterogeneity in the IGF-1 gene rs12423791 recessive model (CC vs. CG+GG), codominant model (CC vs. GG), and all rs6215 comparison models. (Table 2) By the sequential omission of individual studies in these models (C vs. G, CC+CG vs. GG, CG vs. GG), we found that none of the individual studies significantly affected the pooled ORs, and the association between IGF-1 rs12423791 and high myopia did not change, suggesting the high stability of the meta-analysis. The data are not shown but are available upon request.

### Publication Bias

**Fig 6:**
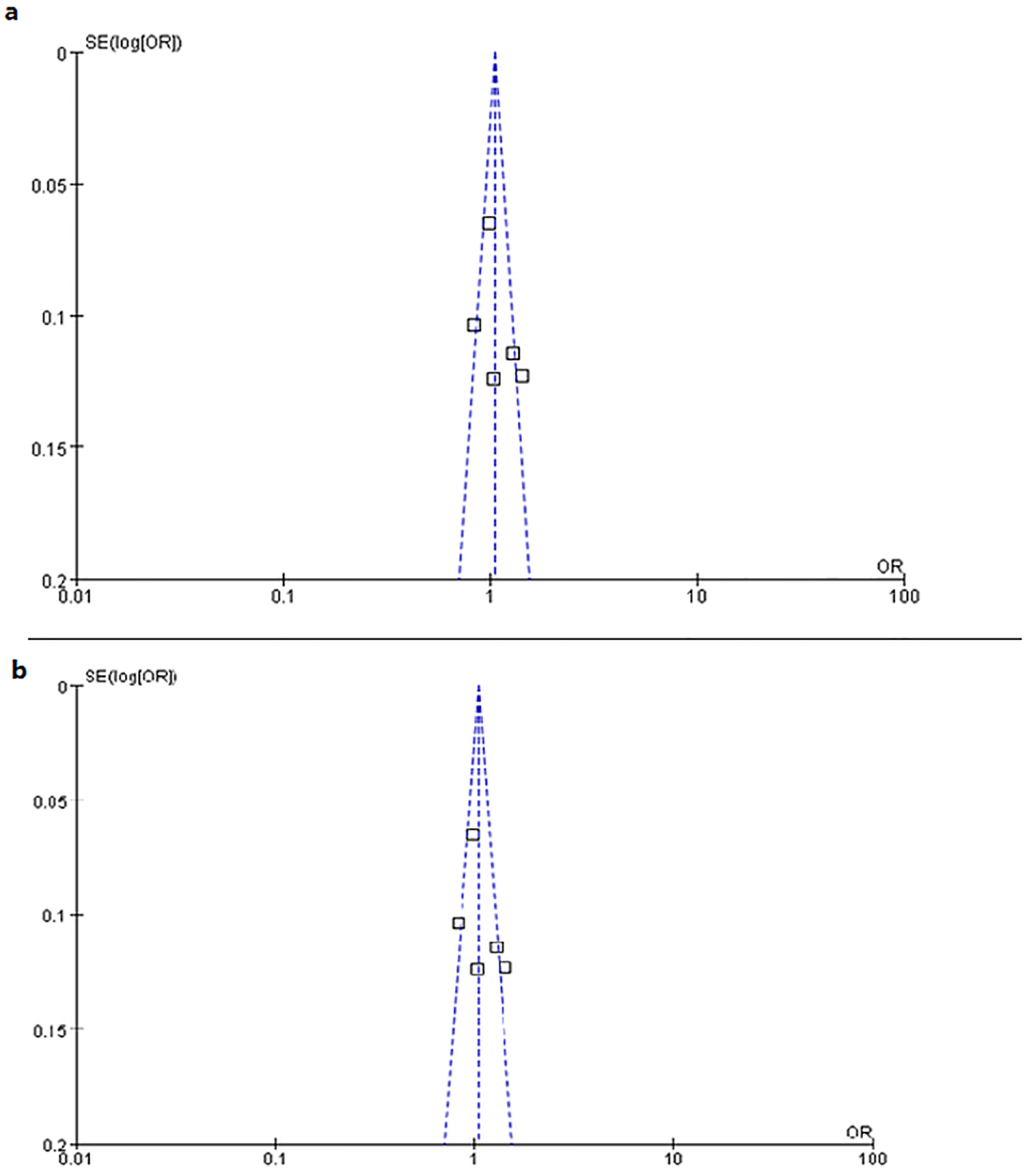
Funnel plot analysis for publication bias. (a) rs12423791 allelic contrast model (C vs. G). (b) rs6214 allelic contrast model (A vs. G).

The shape of the funnel plot did not suggest any obvious asymmetry in allelic contrast models between the rs12423791 and rs6214 polymorphisms and high myopia. (Fig 6) In addition, Egger’s linear regression test on the natural logarithm scale of the OR found no evidence of publication bias for the allelic contrast model (*P*rs12423791 = 0.36, *P*rs6214 = 0.94), the dominant model (*P*rs12423791 = 0.31, *P*rs6214 = 0.84), the recessive model (*P*rs12423791 = 0.71, *P*rs6214 = 0.89), the codominant model of rs12423791 (*P*rs12423791 = 0.45 for CC vs. GG and *P*rs12423791 = 0.29 for CG vs. GG), and the codominant model of rs6214 (*P*rs6214 = 0.95 for AA vs. GG and *P*rs6214 = 0.54 for AG vs. GG). (Table 4)

**Table 4:** Evaluation of publication bias by Egger’s linear regression test.

SNPs	Inherited Model		Coefficient	SE	t	*P*>|t|
**rs12423791**						
	Allelic contrast model	G vs. C	3.04	2.80	1.09	0.36
	Codominant model	CC vs. GG	1.70	1.95	0.87	0.45
		CG vs. GG	2.23	1.75	1.27	0.29
	Dominant model	CC+CG vs. GG	2.62	2.16	1.21	0.31
	Recessive model	CC vs. CG+GG	0.67	1.62	0.41	0.71
**rs6241**						
	Allelic contrast model	A vs. G	-0.08	1.00	-0.09	0.94
	Codominant model	AA vs. GG	0.06	0.88	0.07	0.95
		AG vs. GG	-0.14	0.20	-0.69	0.54
	Dominant model	AA+AG vs. GG	-0.076	0.34	-0.22	0.84
	Recessive model	AA vs. AG+GG	0.21	1.37	0.15	0.89

## Discussion

### Main finding

High myopia is associated with various ocular complications, [31] and it is highly heritable. [32] A genome-wide meta-analysis has provided evidence that several loci that have been shown to be associated with myopia, while the results did not illustrate the influence of IGF-1 gene polymorphisim. [19] To our knowledge, insulin-like growth factor 1 is similar to insulin in function and structure and is a member of a protein family involved in mediating growth and development. [33] The gene encoding insulin-like growth factor 1 is highly conserved between species, and the polypeptide it encodes is insulin-like growth factor 1. [34] As mentioned in the introduction, previous animal studies have demonstrated that insulin-like growth factor 1 contributes to eye growth and myopia development. [22,23] In addition, one study stated that myopia may be related to impaired metabolic control, and the authors observed that enhanced scleral growth may result from increased levels of insulin and insulin-like growth hormones. [35] Recently, genetic studies have indicated that the association between IGF-1 gene rs12423791, rs6214 polymorphisms and the risk of high myopia are not clear. [25,26,27,36,37] Therefore, we performed this meta-analysis to estimate the association between the IGF-1 gene rs12423791 and rs6214 polymorphisms and high myopia. Of the studies included in this meta-analysis, Zhuang et al. [26] demonstrated that IGF-1 rs12423791, but not rs6214, was significantly associated with high myopia in a Chinese population. Mak et al. [36] also reported that a haplotype including rs12423791 was associated with high myopia in another Chinese population. Zhao et al. [37] also reported that rs12423791 but not rs6214 was associated with high myopia in a Chinese population. However, Yoshida et al. [28] reported that IGF-1 rs12423791 was not significantly associated with high myopia in a Japanese population. Similarly, the study by Miyake indicated that none of the tagging SNPs in IGF-1, including rs12423791, were associated with high myopia in a Japanese population. [27] Furthermore, a study in Caucasians which reported that rs6214 in IGF-1 exhibited a significant association with high myopia was not included in this meta-analysis, because it was a family-based design and lacked available data; [25] a cohort study in polish families not finding any significant associations between IGF-1 SNPs (including rs6214) and high myopia, was also excluded in this meta-analysis for its family-based design and only including information about IGF-1 rs6214. [38] The final results of this meta-analysis indicated that rs12423791 or rs6214 was not associated with high myopia in any genetic models. These results were consistent with the recent genome-wide meta-analysis of the high myopia reports of the Consortium for Refractive Error and Myopia (CREAM) which included many ethnicities studies and did not find an association of IGF-1 polymorphisms with myopia. [39,40]

However, our subgroup analysis by study population showed that Chinese populations with the rs12423791 variants may have somewhat higher risks of developing high myopia. Prior researches in China also illustrated that IGF-1 rs12423791 was significantly associated with high myopia in Chinese populations. [26,36,37] As the prevalence of myopia, even high myopia was high in both China and Japan, and the definitions of high myopia and controls in the included studies are similar. [26,27,28,36,37] To reach a definitive conclusion, the results of the study population subgroup analysis should be interpreted cautiously, and more well-designed larger trials using standardized unbiased methods and more ethnic groups should be considered in order to further clarify the association.

To our knowledge, heterogeneity may restrict the interpretation of pooled estimates; therefore, sensitivity meta-analyses were performed in this meta-analysis. We found that none of the individual studies significantly affected the pooled ORs by excluding each study in turn in every comparison, and the association between IGF-1 rs12423791 and high myopia did not change in any genetic models, suggesting the high stability of the meta-analysis. Furthermore, we performed publication bias analyses to validate the reliability of the meta-analysis. In this study, we used a funnel plot and Egger’s linear regression analysis to evaluate for publication bias, and the results showed that there was no evidence of publication bias among the included studies. Therefore, our results suggest that the IGF-1 rs12423791 or rs6214 gene polymorphism was not associated with high myopia susceptibility.

### Limitation

To the best of our knowledge, this is the first meta-analysis to assess the association between rs12423791 and rs6214 of the IGF-1 gene and high myopia. This meta-analysis increases the power to detect and quantify an effect and provides a control for publication differences. Additionally, it must be stressed that there are several limitations to this meta-analysis. First, the study populations were mainly from China and Japan, and study populations from other countries were rare or did not exist. Thus, more studies should be conducted in other ethnic groups to increase the statistical power. Second, the IGF-1 gene is just one of a host of genetic risk factors for high myopia; other genes, such as transforming growth factor β1, may also participate in the pathogenesis of high myopia. Third, our results were based on unadjusted estimates; a more precise analysis should be conducted if individual data are available to allow for adjustment by other covariates, such as age, gender, environmental factors and so on. Finally, this meta-analysis only used the single-marker analysis but not the haplotype analysis for the available data. Despite these limitations, this study also has some merits. First, we did not use the language limitation opinion when searching the literature database to minimize the bias within our research. Additionally, the sensitivity analysis indicated the results of the meta-analysis were stable, and we found no evidence of publication bias among the included studies.

### Conclusion

In summary, this meta-analysis provides evidence that IGF-1 rs12423791 or rs6214 gene polymorphism is not associated with high myopia. Therefore, in view of the fact that myopia is a polygenetic disease, IGF-1 would not play independently important roles in the development of high myopia. Moreover, gene-gene and gene-environment interactions should be considered.

## Supporting Information

S1 Checklist Meta-analysis-on-genetic-association-studies. Meta-analysis on Genetic Association Studies Checklist.
(DOCX)

S2 Checklist PRISMA 2009 Checklist. The PRISMA Checklist for our meta-analysis.
(DOC)
